# Circular RNAs in nucleus pulposus cell function and intervertebral disc degeneration

**DOI:** 10.1111/cpr.12704

**Published:** 2019-10-16

**Authors:** Zheng Li, Xin Chen, Derong Xu, Shugang Li, Matthew T. V. Chan, William K. K. Wu

**Affiliations:** ^1^ Department of Orthopaedic Surgery Peking Union Medical College Hospital Chinese Academy of Medical Sciences and Peking Union Medical College Beijing China; ^2^ Department of Orthopedics The Affiliated Hospital of Qingdao University Qingdao China; ^3^ Department of Anaesthesia and Intensive Care The Chinese University of Hong Kong Hong Kong Hong Kong; ^4^ State Key Laboratory of Digestive Diseases Centre for Gut Microbiota Research Institute of Digestive Diseases and LKS Institute of Health Sciences The Chinese University of Hong Kong Hong Kong Hong Kong

**Keywords:** circRNAs, circSEMA4B, Circular RNA VMA21, CircularRNA_104670, intervertebral disc degeneration

## Abstract

Intervertebral disc degeneration (IDD) is a common cause of low back pain, which inflicts more global disability than any other condition. Although IDD was deemed to be a natural process that comes with ageing, a growing body of evidence suggested that both genetic and environmental factors could modify the development of IDD. In this connection, aberrant function of nucleus pulposus cells has been implicated in IDD pathogenesis. Circular RNAs are a novel class of endogenous non‐coding RNAs that play crucial regulatory roles in diverse cellular processes. Recently, deregulation of circRNAs in nucleus pulposus cells was found to functionally participate in IDD development. In this review, we summarize the current knowledge regarding the deregulation of circRNAs in IDD in relation to their actions on nucleus pulposus cell functions, including cell proliferation, apoptosis and extracellular matrix synthesis/degradation. The potential clinical utilities of circRNAs as therapeutic targets for the management of IDD are also discussed.

## 1 INTRODUCTION

Low back pain is an extremely common condition with an estimated global point prevalence of ~10%, causing distress and economic losses due to pain, activity limitation and work absence. It also ranks highest in terms of overall disability among 291 conditions studied in the Global Burden of Disease 2010 Study.^1^, ^2^, ^3^ Intervertebral disc degeneration (IDD) of the lumbar spine, which is characterized by progressive structural failure and advanced signs of ageing of the intervertebral disc, is strongly associated with an increased risk for low back pain.^4^ Both genetic (eg polymorphisms in genes encoding collagen I, IX, XI and aggrecan) and lifestyle (eg the lack of sports activities and night shift work) factors have been linked to IDD development.^5^, ^6^ However, the exact cellular and molecular mechanisms underlying IDD remain largely elusive. Anatomically, the intervertebral disc consists of a gelatinous core known as nucleus pulposus (NP) surrounded by a lamella of fibrous cartilage termed annulus fibrosus.^7^, ^8^ NP cells play a crucial role in maintaining the integrity of intervertebral discs via producing extracellular matrix (ECM) components, such as aggrecan as well as type II and type X collagen.^9^, ^10^ A growing body of evidence now suggests that aberrant NP cell functions, including altered cell proliferation, apoptosis, ECM production/degradation and cytokine secretion, are key to IDD pathogenesis.^11^, ^12^

Non‐coding RNAs (ncRNAs), including microRNAs (miRNAs), long ncRNAs (lncRNAs) and the recently discovered circular RNAs (circRNAs), are important regulatory elements encoded by the genome and play critical roles in diverse cellular processes.^13^, ^14^ CircRNAs are a type of single‐stranded RNAs that form covalently closed loops that primarily serve as competing endogenous RNAs (ceRNAs) to sponge miRNAs. A subset of circRNAs have also been reported to exert their biological functions via transcription regulation, modulation of alternative splicing, direct interactions with RNA‐binding proteins and protein translation via rolling circle amplification.^15^, ^16^, ^17^ Concerning transcriptional regulation, a class of circRNAs known as exon‐intron circRNAs characterized by circularized exons with introns retained in between has been identified. This class of circRNAs was found to exhibit nuclear localization, interacts with the U1 small nuclear ribonucleoprotein and enhances transcription of their parental genes in a cis‐acting manner.^18^

An accumulating number of studies have demonstrated that circRNAs play pivotal roles in cellular functions such as proliferation, differentiation, invasion, migration and metabolism,^19^ and their deregulation has been documented in different types of diseases, including cancers, neurodegeneration, inflammatory/immune diseases and orthopaedic conditions, such as scoliosis, osteoporosis and osteoarthritis.^20^, ^21^, ^22^, ^23^, ^24^, ^25^ Recently, it has been reported that circRNAs are involved in the development of IDD.^26^ In terms of translational value, intracellular circRNAs are insensitive to exonuclease in contrast with other classes of linear RNAs,^27^ rendering circRNAs suitable for further development into tissue biomarkers owing to their high intracellular stability.

This review serves to provide an overview of the current understanding regarding the functional roles of deregulated circRNAs in modulating phenotypes that are pertinent to IDD pathogenesis, including NP cell proliferation, apoptosis, ECM synthesis/degradation and pro‐inflammatory cytokine production. We also discussed the translational value of circRNAs in terms of their clinical utilities as therapeutic targets for the management of IDD.

## 2 CIRCRNAS EXPRESSION PROFILING IN IDD

Circular RNAs expression profiling with transcriptome sequencing, microarray or PCR array followed by validation with reverse transcription‐quantitative PCR (RT‐qPCR) is the most frequently adopted approach to identify and confirm the differential expression of circRNAs in specific disease states.^20^, ^28^, ^29^ Profiling studies of circRNAs in IDD are listed in Table 1.

**Table 1:** CircRNA expression profiles in intervertebral disc degeneration

No.	Methods	Samples	Microarray filtering criteria	Upregulated	Downregulated	References
1	Microarray RT‐PCR	degenerate disc tissues	fold change more than two and *P* values < .05	354 circRNAs	282 circRNAs	31
2	Microarray RT‐PCR	degenerate disc tissues	*P* < .05; Fold change >2	3724 circRNAs	3570 circRNAs	34
3	Microarray RT‐PCR	degenerate disc tissues	*P* < .05; Fold change >1.5	428 circRNAs	364 circRNAs	35
4	Microarray RT‐PCR	degenerate disc tissues	Fold change >2	51 circRNAs	21 circRNAs	36

Microarray‐based profiling of circRNAs deregulated in IDD was first performed by Liu and colleagues in which they conducted a comprehensive profiling of mRNAs, miRNAs, lncRNAs and circRNAs in five normal discs from cadaveric donors versus five degenerative discs from patients with IDD. The microarray data were then deposited in a public database.^30^ Lan and colleagues conducted an integrative analysis on these datasets with an aim to comprehensively depict the RNA landscape in human IDD.^30^ Among 2894 annotated circRNAs, 636 circRNAs were found to be differentially expressed (354 upregulated and 282 downregulated) in the degenerated discs as compared with normal control discs. Notably, a single miRNA was predicted to interact with a multitude of circRNAs. The upregulation of circRNA‐101852 and downregulation of circRNA‐101645 were confirmed by RT‐qPCR.^31^ Zou and colleagues also utilized the same microarray datasets and identified a total of 76 pairs of differentially expressed circRNAs and their host genes in IDD. Pathway analysis revealed that host genes that encode the upregulated and downregulated circRNAs in IDD are involved in signalling pathways such as Wnt and integrin signalling. Significant upregulation of circ_0008305 and downregulation of circ_0041946 were confirmed in an independent set of human lumbar NP specimens by RT‐qPCR.^32^ Another follow‐up bioinformatic study by Zhang and colleagues using these deposited datasets identified 568 mRNAs, 55 miRNAs, 765 lncRNAs and 586 circRNAs that were significantly differentially expressed in degenerative discs than in normal discs. The authors then reconstructed the ceRNA networks in which three circRNAs, namely circ_0005139, circ_0037858 and circ_0087890, were predicted to be key regulators in IDD progression. The deferentially expressed circRNA circ_0000189 was also predicted to play a central role and have crosstalk with miR‐486‐5p, the lncRNA DANCR and 6 mRNAs—PYCR2 (Pyrroline‐5‐Carboxylate Reductase 2), TOB1 (Transducer of ERBB2‐1), ARHGAP5 (Rho GTPase Activating Protein 5), RBPJ (Recombination Signal Binding Protein For Immunoglobulin Kappa J Region), CD247 (Cluster of Differentiation 247) and SLC34A1 (Solute Carrier Family 34 Member 1).^29^ A recent study by Zhu and colleagues further reconstructed lncRNA/circRNA‐miRNA‐mRNA ceRNA network and identified circRNA_102348, circRNA_102399 and circRNA_100086 as upstream regulators of miR‐185‐5p‐transforming growth factor β1.

(TGFB1)/FOS, miR‐302a‐3p‐hypoxia inducible factor 1 subunit α (HIF1A) and miR‐509‐3p‐mitogen‐activated protein kinase 1 (MAPK1) axes, respectively. The functional synergism between particular circRNAs and lncRNAs [eg circRNA_102348 and metastasis‐associated lung adenocarcinoma transcript 1 (MALAT1)] on downstream miRNA sponging was also depicted.^33^

Aside from these comprehensive datasets produced by Liu et al, other groups of investigators have profiled circRNAs in IDD with their own set of clinical samples. Wang and colleagues isolated and cultured human NP cells from IDD tissues and compared circRNA expression profiles with those from patients suffering from vertebral fracture using microarray.^34^ A total of 7294 circRNAs were found to be deregulated in NP cells isolated from degenerated versus non‐degenerated disc, among which 3570 were downregulated and 3724 were upregulated. The top ten differentially expressed circRNAs were validated by RT‐qPCR. Pathway analysis of the predicted miRNA targets of differentially expressed circRNAs indicated that cell cycle regulation, ubiquitin‐mediated proteolysis and ECM‐receptor interactions in NP cells are implicated in IDD development.^34^ In another study, Song and colleagues performed circRNA microarray with four NP specimens from normal and four from IDD subjects. A total of 792 differentially expressed (428 upregulated and 364 downregulated) circRNAs were identified, among which circRNA_104670 was one of the highest upregulated circRNA and showed extensive interactions with miRNAs. The upregulation of circRNA_104670 was confirmed in an independent set of human NP tissues by RT‐qPCR.^35^ A recent microarray study by Wang and colleagues also reported that human NP cells isolated from degenerated and non‐degenerated discs showed distinct circRNA expression patterns, with 21 downregulated and 51 upregulated by more than twofold in the degenerated NP cells. The upregulation of circ‐4099, circ‐2069, circ‐92328 and circ‐68610 was further confirmed by RT‐qPCR.^36^

The recent advancements in circRNA enrichment and bioinformatic workflows for next‐generation sequencing have facilitated the use of this unbiased genome‐wide approach for the identification of circRNAs. Bioinformatic tools used for predicting and identifying circRNAs have been extensively reviewed by Kulcheski and colleagues.^37^ In this regard, thousands of loci in the human and mouse genomes have been identified to code for circRNAs, which could account for 1% as many molecules as poly(A) RNA. CircRNA isoform splice site selection, the circular to linear isoform ratios, and N^6^‐methyladenosine (m6A) modification of circRNA were all found to be cell type‐specific.^38^, ^39^ It is hopeful that further data mining from existing circRNA microarray datasets together with the application of novel circRNA sequencing approaches could facilitate the identification of key pathogenic circRNAs and potential therapeutic targets in IDD.

## 3 FUNCTIONAL ROLES OF SPECIFIC CIRCRNAS IN IDD

### 3.1 CircSEMA4B

CircSEMA4B (circRNA Semaphorin 4B) was the top downregulated circRNA in human lumbar IDD specimens in Liu et al's^30^ circRNA microarray dataset. Wang et al^26^ further verified circSEMA4B downregulation in an independent set of lumbar IDD samples and correlated its levels with pro‐inflammatory cytokine and ECM component expression. They found that circSEMA4B levels were negatively correlated with interleukin (IL)‐1β and tumour necrosis factor (TNF)‐α but positively with aggrecan and collagen II mRNA expression. In cultured NP cells, IL‐1β downregulated circSEMA4B expression, suggesting that IL‐1β was upstream of circSEMA4B. Functionally, IL‐1β lowered collagen II and aggrecan expression, reduced the viability and induced the senescence of NP cells, all of which were reversed by the enforced expression of circSEMA4B. Consistently, knockdown of circSEMA4B aggravated these pathogenic phenotypes in the presence of IL‐1β. Mechanistically, the protective phenotypes produced by enforced expression of circSEMA4B disappeared upon pharmacological Wnt signalling activation. Concordantly, circSEMA4B was found to function as a molecular sponge for reducing the availability of miR‐431 to derepress the expression of glycogen synthase kinase (GSK)‐3β and secreted frizzled‐related protein 1 (SFRP1),^26^ both of which are known upstream repressors of the Wnt pathway.^40^, ^41^ These findings collectively indicate that circSEMA4B could antagonize the pro‐IDD Wnt signalling through the miR‐431‐GSK‐3β/SFRP1 axis in NP cells. Restoring circSEMA4B expression or antagonizing miR‐431 might thus represent a novel therapeutic approach for rectifying IL‐1β‐triggered degenerative processes.

### 3.2 CircRNA_104670

Song et al^35^ performed circRNA microarray and identified circRNA_104670 as one of the highest upregulated circRNAs in degenerative NP tissues. Bioinformatic reconstruction of the regulatory network of circRNA_104670 revealed that this circRNA might act through the miR‐17‐3p‐matrix metallopeptidase (MMP)‐2 axis in NP cells. Consistently, upregulation of circRNA_104670 and downregulation of miR‐17‐3p in degenerative NP tissues were confirmed by RT‐qPCR and were found to be associated with Pfirmman scores (a magnetic resonance imaging‐based disc degeneration grading system). Luciferase and EGFP/RFP reporter assays further verified circRNA_104670 as a sponge for miR‐17‐3p to upregulate MMP‐2 (a collagen‐degrading MMP). Functionally, circRNA_104670 promoted apoptosis and inhibited proliferation of NP cells and repressed collagen II expression. Importantly, the pro‐IDD effect of circRNA_104670 was confirmed in vivo in which mice injected with small interfering‐RNA (siRNA) against circRNA_104670 delivered via adeno‐associated virus exhibited lower IDD grades. The in vivo protective action of circRNA_104670 knockdown was also attenuated upon miR‐17‐3p inhibition.^35^ These findings suggest that circRNA_104670 contributes to IDD development through impairing NP cell survival and shifting the balance towards ECM degradation via sponging miR‐17‐3p.

### 3.3 Circ‐4099

Circ‐4099, located on exonic chromosome 11 and aligned in the sense direction of the protein‐coding gene *DENND5A* (DENN domain containing 5A), was one of the top upregulated circRNAs in Wang et al's^36^ microarray study. RT‐qPCR and luciferase reporter assay further revealed that circ‐4099 transcription could be induced by TNF‐α in human NP cells, where such induction was blocked by mitogen‐activated protein kinase (MAPK) and nuclear factor (NF)‐κB pathway inhibitors. Enforced expression of circ‐4099 also enhanced aggrecan and collagen II but reduced TNF‐α and IL‐1β expression in NP cells. Mechanistically, circ‐4099 serves as a sponge for miR‐616‐5p as confirmed by immunoprecipitation for AGO2 (Argonaute RNA‐Induced Silencing Complex [RISC] Catalytic Component 2) and RNA–RNA pull‐down assay. The upregulation of aggrecan and collagen II and downregulation of TNF‐α and IL‐1β were also reversed by miR‐616‐5p mimics. Sox9 (SRY‐Box 9), a transcription factor that promotes the expression of chondrocyte‐specific genes including aggrecan and collagen II,^42^, ^43^ was confirmed to be the direct target of miR‐616‐5p.^36^ These findings suggest that upregulation of circ‐4099 in NP cells could act as an inflammation‐responsive autoprotective mechanism against IDD development via modulating the miR‐616‐5p‐Sox9 pathway.

### 3.4 Circ‐GRB10

Guo and colleagues reanalysed Liu et al's^30^ microarray datasets and showed that circ‐GRB10 (Growth Factor Receptor‐Bound Protein 10) and miR‐328‐5p were one of the most significantly negatively correlated circRNA‐miRNA pairs.^44^ By RT‐qPCR, the investigators verified the significant downregulation of circ‐GRB10 and upregulation of miR‐328‐5p as well as their negative correlation in an independent set of IDD NP samples. Functionally, enforced expression of circ‐GRB10 suppressed NP cell apoptosis whereas knockdown of circ‐GRB10 produced the opposite the effect, suggesting that the aberrant downregulation of circ‐GRB10 in NP tissues might contribute to IDD through impairing NP cell survival. Mechanistically, reduced circ‐GRB10 levels were found to increase the availability of miR‐328‐5p and thereby repressing ERBB2 (v‐erb‐b2 erythroblastic leukaemia viral oncogene homolog 2; a target of miR‐328‐5p). These data suggested that deregulation of circ‐GRB10/miR‐328‐5p/ERBB2 pathway was involved in IDD development.^44^ Restoring the expression of circ‐GRB10 or enhancing the ERBB2 signalling might thus serve a new therapeutic approach for the IDD.

### 3.5 CircVMA21

Cheng et al^45^ showed that miR‐200c was overexpressed in human IDD NP samples and inducible by TNF‐α plus IL‐1β in cultured NP cells where enforced expression of miR‐200c promoted apoptosis and shifted ECM homeostasis from anabolism towards catabolism (ie reduced aggrecan and collagen II but enhanced ADAMTS (A Disintegrin and Metalloproteinase with Thrombospondin motifs)‐4, ADAMTS‐5, MMP‐13 and MMP‐3 expression). miR‐200c mediated these biological functions in NP cells via suppressing the expression of X linked inhibitor of apoptosis protein (XIAP). In this regard, circVMA21 (circRNA Vacuolar ATPase Assembly Factor VMA21) was found to be downregulated in IDD NP tissues, where it served as a sponge for miR‐200c and restrained apoptosis and ECM anabolism/catabolism imbalance induced by TNF‐α and IL‐1β. Importantly, the authors showed that intradiscal injection of adenoviral circVMA21 alleviated IDD in rats. These data demonstrated that aberrant downregulation of circVMA21 contributed to IDD phenotypes via the miR‐200c/XIAP axis. Restoring circVMA21 expression might thus be a viable approach for attenuating NP cell apoptosis and reversing ECM anabolism/catabolism imbalance against IDD development.

## 4 CONCLUSION AND FUTURE PERSPECTIVES

**Figure 1:**
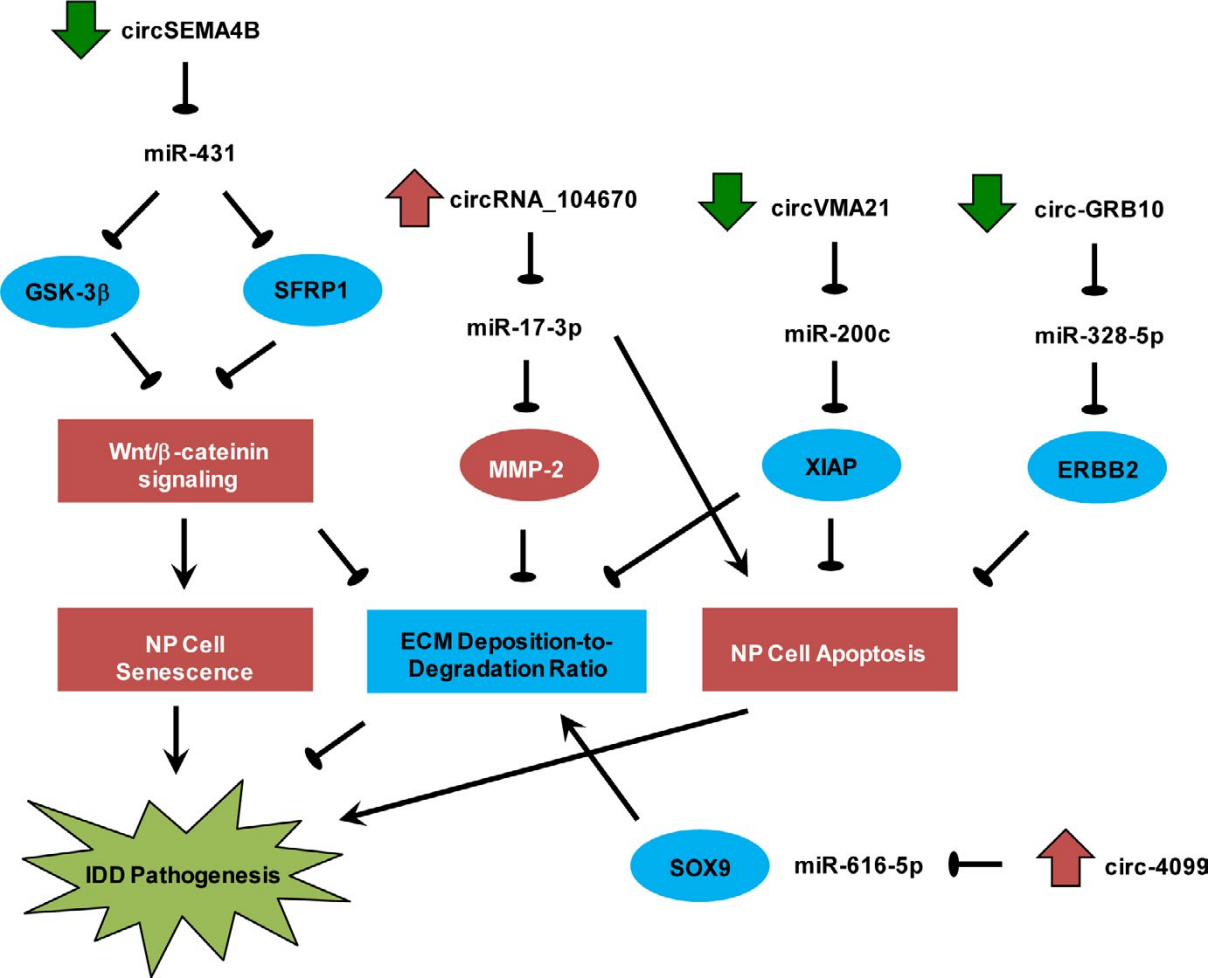
Upregulation of circRNA_104670 and downregulation of circSEMA4B, circ‐GRB10 and circVMA21 contribute to IDD pathogenesis through promoting NP cell apoptosis/senescence, enhancing ECM degradation or reducing collagen II/aggrecan synthesis. Upregulation of circ‐4099 represents an autoregulatory protective response against IDD development

Intervertebral disc degeneration is a common cause of low back pain which is a serious public health issue. Previous studies have shown that aberrant NP cell functions, including proliferation, apoptosis, senescence, ECM deposition/degradation and cytokine secretion, are causally involved in IDD pathogenesis. Recently, it was shown that two classes of ncRNA, namely miRNAs and lncRNAs,^11^, ^12^ play key roles in modulating NP cell phenotypes during IDD development. Emerging evidence, as summarized by our review, also indicated that deregulation of circRNAs, a newly reported class of ncRNA, was involved in IDD development (Table 2). Deregulation of these circRNAs (eg circSEMA4B, circRNA_104670, circ‐4099, circ‐GRB10 and circVMA21) modulates the aforementioned NP cell phenotypes through sponging their target miRNAs and thereby repressing or derepressing the corresponding downstream mRNAs (Figure 1). Treatment for IDD might thus be achieved through restoring the expression of downregulated circRNAs or silencing of the aberrantly upregulated circRNAs. Nevertheless, the best way to achieve NP cell‐specific delivery of circRNA‐based therapeutics remains undefined. The relative importance of different classes of ncRNAs (ie miRNAs, lncRNAs and circRNAs) in IDD development is also unclear, rendering prioritization and selection of therapeutic targets difficult. Aside from therapy, early detection and prognostication of IDD are clinically challenging. Due to the tissue‐ and development stage‐specific expression pattern of circRNAs, their use as biomarkers has been promulgated. In this connection, the use of circulating or tissue circRNAs for diagnosing or prognosticating diseases, including cancers, cardiovascular diseases, diabetes, autoimmune diseases and infections, has been demonstrated.^46^ However, such endeavours have not been attempted in IDD. More translational work, for example, systematic identification and multi‐cohort validation of circulating circRNAs associated with disease status, clinical outcomes and treatment responses in IDD patients, is thus required to maximize the clinical utilities of circRNAs for the management of IDD.

**Table 2:** Functional characterization of the circRNAs in intervertebral disc degeneration

CircRNAs	Deregulation	Functional roles	Related genes/pathways	References
CircSEMA4B	Downregulated	Modulating inflammatory cytokine and matrix metalloproteinase expression	Wnt miR‐431 GSK‐3β SFRP1	26
CircRNA_104670	Upregulated	Cell proliferation, ECM degradation	miR‐17‐3p MMP‐2	35
Circ‐4099	Upregulated	Inflammation, ECM degradation	MAPK NF‐κB miR‐616‐5p Sox9	36
Circ‐GRB10	Downregulated	Apoptosis	miR‐328‐5p ERBB2	44
CircVMA21	Downregulated	Apoptosis, Inflammation	miR‐200c XIAP	45

## Data Availability

Research data are not shared.
